# Exploring the worldwide impact of COVID-19 on conflict risk under climate change

**DOI:** 10.1016/j.heliyon.2023.e17182

**Published:** 2023-06-10

**Authors:** Xiaolan Xie, Mengmeng Hao, Fangyu Ding, Tobias Ide, David Helman, Jürgen Scheffran, Qian Wang, Yushu Qian, Shuai Chen, Jiajie Wu, Tian Ma, Quansheng Ge, Dong Jiang

**Affiliations:** a Institute of Geographic Sciences and Natural Resources Research, Chinese Academy of Sciences, Beijing, 100101, China; b College of Resources and Environment, University of Chinese Academy of Sciences, Beijing, 100049, China; c Murdoch University, Murdoch, 6150, Perth, Australia; d Institute of Environmental Sciences, Department of Soil and Water Sciences, The Robert H. Smith Faculty of Agriculture, Food & Environment, The Hebrew University of Jerusalem, Rehovot, 7610001, Israel; e Advanced School for Environmental Studies, The Hebrew University of Jerusalem, Jerusalem, Israel; f Institute of Geography, Center for Earth System Research and Sustainability, University of Hamburg, Hamburg, 20144, Germany; g Centre for Tropical Medicine, Nuffield Department of Clinical Medicine, University of Oxford, United Kingdom

**Keywords:** COVID-19, Conflict risk, Causal link, Structural equation model, Boosted regression trees

## Abstract

**Objectives:**

Understand whether and how the COVID-19 pandemic affects the risk of different types of conflict worldwide in the context of climate change.

**Methodology:**

Based on the database of armed conflict, COVID-19, detailed climate, and non-climate data covering the period 2020–2021, we applied Structural Equation Modeling specifically to reorganize the links between climate, COVID-19, and conflict risk. Moreover, we used the Boosted Regression Tree method to simulate conflict risk under the influence of multiple factors.

**Findings:**

The transmission risk of COVID-19 seems to decrease as the temperature rises. Additionally, COVID-19 has a substantial worldwide impact on conflict risk, albeit regional and conflict risk variations exist. Moreover, when testing a one-month lagged effect, we find consistency across regions, indicating a positive influence of COVID-19 on demonstrations (protests and riots) and a negative relationship with non-state and violent conflict risk.

**Conclusion:**

COVID-19 has a complex effect on conflict risk worldwide under climate change.

**Implications:**

Laying the theoretical foundation of how COVID-19 affects conflict risk and providing some inspiration for the implementation of relevant policies.

## 1 Introduction

As the biggest threats facing mankind, COVID-19 and conflict risk severely affected human livelihood and economic development. Exploring the effects of COVID-19 on conflict risk is essential to human health and safety. However, no clear conclusion has been drawn from previous studies regarding the causal link between COVID-19 and conflict risk, which limit the implementation of corresponding prevention policies. To address this knowledge gap, this study applies multiple approaches to understand if and how COVID-19 affects multiple types of conflict worldwide.

### 1.1 Background

The adverse effect of COVID-19 on social stability has unfolded over recent years as the virus spread across the world, stemming from the drastic alteration of human lifestyles and the disservice this pandemic has done to the healthcare and economic systems [1,2]. This huge health hazard to the world has been escalating while generating broad-chain effects from severe recession to open social protest [[3], [4], [5]], all of which ignited concern that COVID-19 might affect conflict worldwide [[6], [7], [8]].

Since the COVID-19 induced recession has resulted in many individuals losing their jobs, the epidemic increased the likelihood that people may join violent groups because the groups pay their members [[9], [10], [11], [12]]. Furthermore, the populace becomes increasingly frustrated with the lack of governmental responses to COVID-19 while the security forces are overstretched to enforce lockdown orders that restrict nonessential commercial activities and industries to manage the spread of the virus [13,14]. More individuals are incited to join the opposition movement to vent their grievances, bringing up the potential for flare-up violence [15]. Another unintended consequence of this shortage in security forces is the asymmetrical advantage for rebels groups challenging the state. This is particularly acute as the state also faced considerable financial constraints due to the COVID-19 induced recession [16,17]. COVID-19 also constrains peacebuilding operations due to lockdowns, gathering restrictions, and funding shortages [18].

### 1.2 Related research

Despite the increasing evidence for the view that the COVID-19 restrictions and their subsequent economic fallout might affect conflict dynamnics [19,20], this perspective is hardly backed by empirical research because all the existing studies are descriptive except for a few research studies making limited statistical analyses. For example, Ide [7] analyzed battles and explosion events per month for nine countries in the first months of the COVID-19 pandemic. Bloem and Salemi [8] examined trends of daily event counts for different types of conflict preceding and following the COVID-19 pandemic. Grasse et al. studied the patterns of state violence against civilians in Africa during the COVID-19 related lockdowns [21].

However, given the complexity of the effect of COVID-19 on conflict, descriptive and limited statistical analysis using only conflict and COVID-19 data might be inadequate to yield persuasive results as they can omit critical information regarding, for example, the role of climate [22,23]. Moreover, since the effect of COVID-19 on conflict risk might vary between regions and types of conflict, it is doubtful that the research focusing on a single type of conflict or specific countries could comprehensively capture the combined effects of COVID-19 on multiple types of conflict worldwide.

### 1.3 Theoretical framework

Conflict is a complex and dynamic social phenomenon whose onset, intensity, and duration are driven by various factors. To better explore the effects of COVID-19 on conflict, we must dig into previous research on the relationship between COVID-19 and conflict and their respective fields in general [24]. We found earlier studies focusing on onclimate impacts conflict risk that successfully incorporated environmental background data into a climate-conflict modeling framework [[25], [26], [27], [28]]. When adding COVID-19 as a factor in such a modeling framework, there appears to be a consistent link between COVID-19 spread and conflict. Moreover, the distribution pattern of such a link has shown some connection with climate, which has driven us to examine the climate-COVID-19-conflict nexus [29,30].

The climate-COVID-19-conflict nexus is complex that cannot be easily captured by statistical analyses. Reading through pertinent literature, we found that Structural Equation Modeling (SEM) is most appropriate for this study because it has been widely used in exploring the complex nexus between multiple variables [31]. When proposing hypotheses about how climate, conflict risk, and COVID-19 interact, we can use SEM to test the relationships with real data, thus helping us to understand if and how COVID-19 affects multiple types of conflict worldwide under climate change. In this study, we assumed that (1) temperature and precipitation have not only direct causal effects on conflict but also indirect links to conflict mediated through COVID-19, and (2) COVID-19 has impacts on both current and one-month lag conflict. In addition, we also included the environmental background data such as population density, urban accessibility, and infant mortality rate in SEM.

### 1.4 Objectives

This study aimed to capture the underlying effect pattern of COVID-19 (deaths per month) on conflict risk (the incidence of non-state armed conflict, violent conflict, and demonstrations) under climate change (temperature, precipitation), detailed information on these datasets is provided in the Data and Table S1. Our ultimate research goal is to comprehensively examine the effects of COVID-19 on three types of conflict in six geographical regions (sub-Saharan Africa, South Asia, Middle East, South America, North America, and Europe), thereby providing insights into health preventive and conflict risk early warning.

## 2 Materials and methods

### 2.1 Structural Equation Modeling

Structural Equation Modeling (SEM) is a widely used multivariate statistics method, which encompasses factor analysis and path analysis [32]. Based on proposed hypotheses about how each variable works, SEM formalizes the structural correlation among cause-and-effect variables into an equation system by evaluating the direct and indirect effects of each predictor variable simultaneously [33]. We performed the SEM to test the links between climate, COVID-19, and three types of conflict both globally and regionally. The model was conducted in IBM SPSS AMOS Version 21 using a bootstrap procedure (2000 replications) to estimate the standard errors of the coefficients for the final model at the 95% confidence interval.

We calculated the tolerance and variance inflation factor (VIF) of the data based on IBM SPSS Statistics 24 to assess the collinearity of the variables before fitting SEM (Table S2). Results indicated that the collinearity of variables is within the acceptable range of the SEM model. Five indices of model goodness fit were set with conventional significance thresholds: the comparative fit index (CFI > 0.9), the norm fit index (NFI > 0.9), the goodness-of-fit index (GFI > 0.9), the standardized root mean square residual (SRMR < 0.1), and the root mean square error of approximation (RMSEA < 0.1) —to evaluate the overall fitness of each SEM. The greatly fitting statistical results suggest the good performance of all indexes in each SEM, meaning that the model functions well in describing the data (Tables S3–9).

### 2.2 Boosted Regression Tree

Boosted Regression Tree (BRT) is an ensemble machine learning method for fitting statistical models [34]. Unlike traditional regression methods that produce one single best model, BRT combines the advantages of regression trees and boosting, making an optimized performance of prediction through the technique of boosting and minimizing the prediction error by the progressive addition of regression trees, thusly rendering it a suitable tool to simulate the complex conflict risk [34]. We took COVID-19, climate, and environmental background variables as independent variables, while the conflict was the dependent variable, using the BRT model to simulate the conflict risk.

Based on RStudio Version 3.3.3 statistical programming environment, the ‘dismo’ and ‘gbm’ packages were adopted to deploy the BRT modeling framework. In this study, the main tuning parameter values were set as follows: (tree.complexity = 4, learning.rate = 0.01, bag.fraction = 0.75, step.size = 10, cv.folds = 10, max.trees = 10,000). In addition, an ensemble of 50 BRT models was built to enhance the robustness of the modeling approach. A tenfold cross-validation area under the receiver operating characteristic curve (ROC-AUC) was adopted as an accuracy statistics index to avoid over-fitting.

To assess the impact of COVID-19 on the conflict simulation, we set strategy A and strategy B in the BRT modeling. Specifically, we set COVID-19 as an independent variable in strategy A, while excluding it in strategy B to verify whether COVID-19 affects the predictive accuracy in conflict simulation.

**Table:** 

strategy A	strategy B
**COVID-19**	
Temperature	Temperature
Precipitation	Precipitation
Water resources	Water resources
Crop yield	Crop yield
Land cover	Land cover
Population density	Population density
Infant mortality rate	Infant mortality rate
Altitude	Altitude
Gross cell product	Gross cell product
Ethnic Power Relations	Ethnic Power Relations
Urban accessibility	Urban accessibility

## 3 Data

To fully estimate the influence of COVID-19 on different conflict types worldwide, we used data acquired from UCDP GED version 21.1 (Uppsala Conflict Data Program Georeferenced Event Dataset) [35] and ACLED (Armed Conflict Location and Event Dataset) [36] datasets to gather spatial coordinates (longitude and latitude) and temporal information of conflict that occurred in 2020–2021 globally.

The UCDP dataset is a joint project of the Center for the Study of Civil War at the Peace Research Institute Oslo. It is defined as an event where an organized actor used armed force against another organized actor or civilians, resulting in at least one direct death at a specific location on a specific date [35]. According to previous research [26], small-scale conflicts are more likely to be sensitive to environmental and climatic changes than large-scale ones. Thus, we exclude all state conflict events that are relatively long-term, which are mainly the conflict events where both the actor and targets are states or governments, and focus on the non-state conflict in which at least one of the warring parties is not a state or government.

The ACLED data collected the dates, actors, locations, and fatalities of all reported violent and non-violent actions across most countries worldwide, which allowed the localized study of conflict within a country’s borders [37]. The database distinguishes between various types of violence (violent events, demonstrations, and non-violent actions) [36]. Here we focus on violent events (battles, explosions/remote violence, and violence against civilians) and demonstrations (protests and riots) to estimate the effect of COVID-19.

To generate monthly conflict data in 2020–2021 with a spatial resolution of 0.5°, we defined one month as the basic time unit to conduct statistics on conflict data before assigning every conflict event to the grid with a cell size of 0.5 × 0.5°. We converted the number of events in each grid cell to binary values of 0 and 1, where 1 indicated experienced conflict during this year, otherwise, 0 is assigned.

(1) $${Conflict\;Risk}_{t}=\left\{ \begin{array}{c} 1\;if\;a\;conflict\;event\;occurs\;in\;month\;t\\ 0\;if\;no\;conflict\;event\;occurs\;in\;month\;t \end{array}\right.$$

Based on previous research, we collected data on climate, agriculture, disease, social, political, economic, and environment that may be associated with conflict [37,38]. With the data acquired, we classified all variables into three categories.

First, we used the death toll as an index to measure the pandemic impact with the COVID-19 data obtained from the Center for Systems Science and Engineering (CSSE) at Johns Hopkins University [39].

Second, in recent years, the research linkages between climate and conflict have been intensively studied [[25], [26], [27]]. Considering that previous studies have indicated the possible complicated direct causal effects of climate on conflict [37], we included temperature and precipitation as variables that characterize the climatic impacts. Their monthly data from 2020 to 2021 were extracted from the reanalysis data (ERA5-Land) [40].

Third, we identified the environmental background variables (control variables) that may determine regional differences that lead to the inconsistency of conflict incidence across regions. Some studies suggested that water stress and food shortage may increase the chance of conflict [22].To measure water stress, we obtained monthly soil moisture data from the National Aeronautics and Space Administration (NASA) Goddard Earth Sciences Data Information Services Center (GES DISC). To quantify changes in crop yield, we used the monthly Normalized Difference Vegetation Index (NDVI) from the Aqua Moderate Resolution Imaging Spectroradiometer (MODIS). Previous research suggested that high population density and ease of traveling might be associated with more conflict, and they are usually used as predictors of conflict [41]. To capture the population and communicative value of a location, we included the population density of 2020 derived from Gridded Population of the World (GPW v4) [42] as well as accessibility to high-density urban centers for 2015 [43]. Some studies have indicated a link between low socioeconomic status and conflict. Since the standard measure of income levels (Gross Domestic Product per capita) at the grid-cell level are currently unavailable, we instead used the infant mortality rate of 2015 obtained from the Socioeconomic Data and Applications Center [44], and gross cell product (USD) of 2005 in the Geographically based Economic data (G-Econ) dataset v4.0 [45] to estimate the socioeconomic status and dynamics. The extent of agricultural dependence and geographical advantage contributes to the inconsistent response of organized actors on conflict [27,46]. To capture a geographical advantage and preferences of conflict, we included the land cover of 2009 from GlobCover and altitude data obtained from NASA Shuttle Radar Topography Mission (SRTM) [46]. We also included ethnic diversity at a grid-cell level from a geocoded version of the Ethnic Power Relations dataset (Geo**E**PR) [47] given that violent conflicts are more likely to take place in regions where ethnic groups are marginalized [48] (Table S1).

After obtaining the dataset of COVID-19, climate change, and environmental background variables, we used ArcGIS Version 10.5 to convert all data into grid data of 0.5°, allowing all data to be modeled on a single spatiotemporal scale.

## 4 Results

### 4.1 Evaluating the links between COVID-19, climate, and conflict

**Fig. 1:**
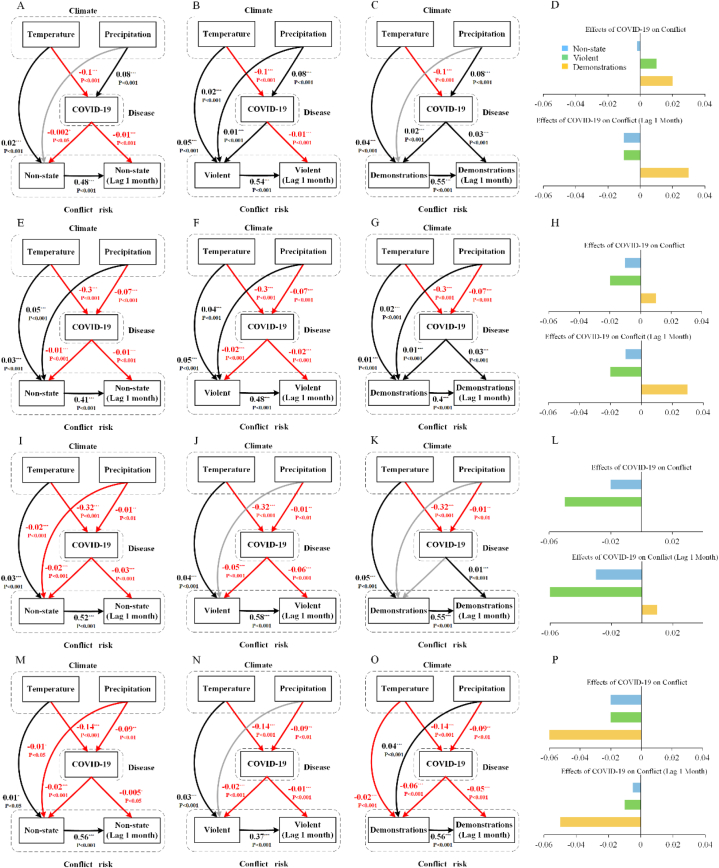
Structural Equation Model showing the link between climate, COVID-19, and three types of conflict at the global scale (A–D) and in sub-Saharan Africa (E–H), North America (I–L), and Europe (M–P). The number next to the arrows indicates the standardized effect. The red, black, and gray lines indicate negative, positive, and not significant effects, respectively, in the model. (For interpretation of the references to color in this figure legend, the reader is referred to the Web version of this article.)

Following tests of collinearity and reliability for SEM (see Supplemental material and Tables S2–9 for summary statistics and parameter estimates), we partitioned and quantified the links between COVID-19, climate, and environmental background factors and their single and combined effects on three categories of conflict across the world. We refer here to conflict as the incidence of single or multiple conflict events occurring during a timescale of one month. Globally, our results suggest that temperature had a negative direct impact on COVID-19 (standardized effect: V = −0.1), which means that COVID-19 was more widespread in colder regions. At the same time, we found a positive impact of precipitation on COVID-19 (V = 0.08), meaning that rainy areas were more prone to COVID-19 spread (Fig. 1A–D). Regionally, however, there was a geographical variation in the pattern of the climatic effects of COVID-19 (Table S10, Fig. 1, and Figs. S1–3). For example, both temperature and precipitation negatively affected COVID-19 transmission in sub-Saharan Africa (Fig. 1E–H), North America (Fig. 1I–L), and Europe (Fig. 1M–P), while in South Asia (Fig. S1) both temperature (V = 0.24) and precipitation (V = 0.09) had a positive impact on COVID-19.

Under such complex climate influences, COVID-19 affected three kinds of conflict types on a global scale (Table 1, Fig. 1, and Figs. S1–3). Our SEM results indicate that COVID-19 had a marginally-negative effect (V = −0.002) on non-state conflict while the impact of COVID-19 on the incidence of violent events (V = 0.01) and demonstrations (V = 0.02) was primarily positive. Regionally, the impact of COVID-19 on non-state conflict risk is consistently negative in all regions. COVID-19 has negative effects on violent conflict risk in all regions but in South America where the presence of COVID-19 increased the violent conflict risk (V = 0.04). There was a positive impact of COVID-19 on the incidence of demonstrations in sub-Saharan Africa (V = 0.01) and the Middle East (V = 0.04) while in South Asia (V = −0.03), South America (V = −0.04), and Europe (V = −0.06) the effect of COVID-19 was negative. Only in North America, the COVID-19 effect on the incidence of demonstrations was insignificant (P = 0.132). In addition, COVID-19 showed to have a lag effect (one-month lag) by decreasing the incidence of non-state and violent conflict while increasing that of demonstrations in sub-Saharan Africa, South Asia, the Middle East, and North America. The effects of all the other influencing variables on the three conflict types are presented in Tables S11–17.

**Table 1:**
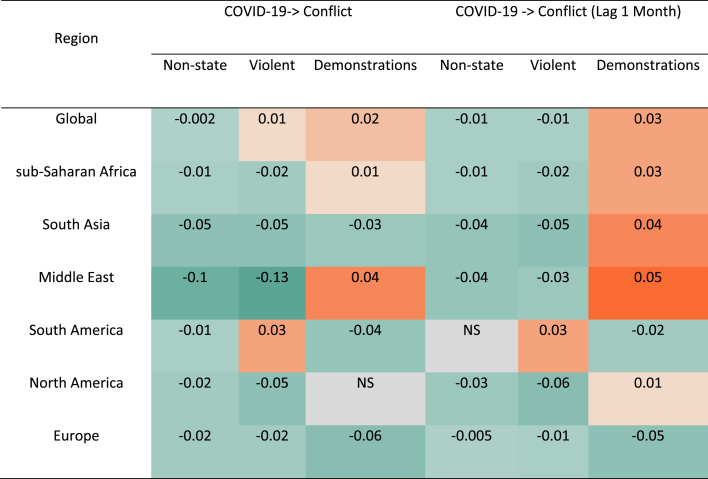
Summary of the influence of COVID-19 on three types of conflict at global and regional scales. NS indicates non-significant (P > 0.05), red indicates positive, and green indicates negative effect. The results of importance in the SEM model are sorted by the shade of color.

In general, the SEM results confirm the hypothesized pathways in our conceptual model (Fig. 1), revealing the complex links between climate, COVID-19, and the incidence of three types of conflict. The quantitative description of each relationship shows that the effect of COVID-19 on the three types of conflict varies at global and regional scales. This allows us to move significantly beyond previous studies where only a single type of conflict in a specific region was assessed, thereby preventing a generalized conclusion of the COVID-19 effect on conflict risk. It is also worth noticing that we obtain roughly consistent results of the one-month lagged effects of COVID-19 on conflict risk, both globally and regionally. This finding might validate a certain time lag of the impact of COVID-19 on conflict risk, demonstrating the significance of considering a time lag to understand the pattern of effects better.

### 4.2 Estimating the effect of COVID-19 on conflict

**Fig. 2:**
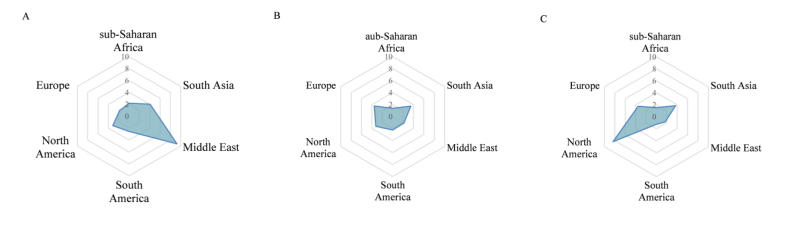
The average importance of COVID-19 on non-state conflict (A), violent conflict (B), and demonstration (C) across all regions.

We used the BRT approach to quantify the impact of COVID-19 on the incidence of conflict and to simulate conflict risk. Our results show that the simulated average importance of COVID-19 in explaining the three types of conflict across all regions ranges from 1.27% to 9.27% (Fig. 2). This means that COVID-19 had a non-negligible contribution to conflict risk in the last two years, which varied by region and the type of conflict. COVID-19 had the highest contribution to non-state conflict (Fig. 2A) and demonstrations (Fig. 2C) in the Middle East and North America, respectively, while its contribution to the incidence of violent conflict (Fig. 2B) was relatively consistent across all regions (Figs. S4–6 shows the average importance of other variables on conflict risk).

**Fig. 3:**
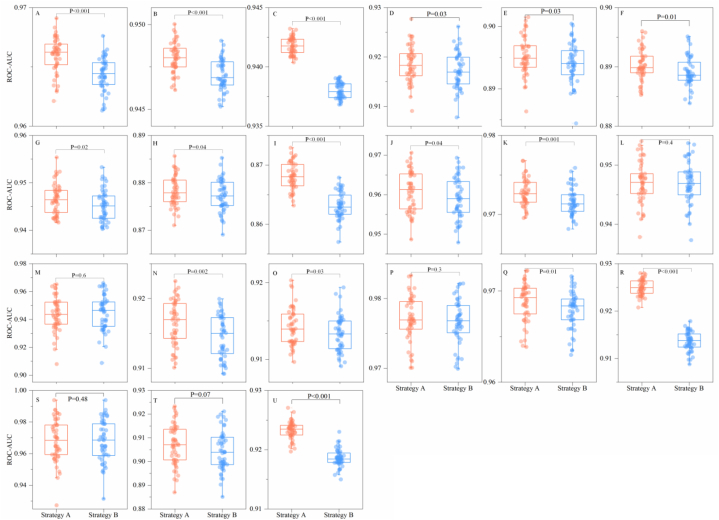
ROC-AUC boxplots of 50 BRT models for two strategies (strategy A and strategy B) assessing the impact of COVID-19 on non-state conflict, violent conflict, and demonstrations in global (A–C), sub-Saharan Africa (D–F), South Asia (G–I), Middle East (J–L), South America (M–O), North America (P–R) and Europe (S–U), respectively. Plots show the median, first and third quantiles, and upper and lower bounds. Wilcoxon test and *t*-test are used in the univariate comparison between two strategies where P < 0.05 is considered statistically significant, while 0.05 < P < 0.1 was considered marginally significant.

To further test whether our simulated results from the SEM models are consistent with the observations, we set up two contrasting strategies using the BRT model, which is based on an ensemble of machine learning algorithms. In the first strategy, we included COVID-19 as an independent variable in BRT (strategy A), while in the second strategy, we excluded COVID-19 from the BRT model (strategy B). By comparing the simulation accuracy (ROC-AUC, area under the receiver operating characteristic curve) of the two strategies, we were able to confirm whether our SEM results are reliably simulating COVID-19 impacts on conflict risk. Indeed, this independent approach corroborated the significant impact of COVID-19 on the three types of conflict worldwide (Fig. 3).

The predictive performance of strategy A was significantly higher than that of strategy B for non-state violence, violent conflict, and demonstrations when analyzing data globally (Fig. 3A–C). However, there were clear differences among the regions in terms of the COVID-19 effect on the different conflict types (Fig. 3D–U). For example, COVID-19 was a significant factor in explaining the incidence of demonstrations in sub-Saharan Africa (Fig. 3F), South Asia (Fig. 3I), and Europe (Fig. 3U), and especially in North America (Fig. 3R) while in the Middle East (Fig. 3K) and South America (Fig. 3N) there was a stronger effect of COVID-19 on the incidence of violent conflict. In some cases, for example in Europe (Fig. 3S–U), there was no clear contribution of COVID-19 to some of the forms of conflict, which likely demonstrates the interactive nature of COVID-19 and other factors on the effect on human behavior.

## 5 Discussion

This study assessed the effect of COVID-19 on three types of conflict risk across the world and six regions. Our contribution to COVID-19-conflict nexus research was primarily manifested in three aspects: (1) By incorporating COVID-19 into methods derived from earlier conflict studies, we were able to separate the effect of COVID-19 on conflict risk from the effect of climate on COVID-19 and the indirect effect of climate on conflict caused by COVID-19. (2) Our results suggest that COVID-19 has a significant impact on conflict risk, showing consistency with some previous studies [6,7]. However, in contrast to earlier studies that only examined the direct impact of COVID-19 on conflict, our research could more accurately estimate and model the effect of COVID-19 on conflict along with a variety of other conflict-driving factors. (3) Our findings concur with the sensitivity of COVID-19 to the climate discovered in earlier studies [30] in terms of how the climate affects it. Other than the positive effect of temperature on COVID-19 in South Asia and South America (two regions that are predicted to get hotter under climate change), we observed that cold weather is to a certain degree associated with increased COVID-19 transmissibility in some regions [29].

While recognizing the non-negligible effect of COVID-19 on conflict risk at the global scale, our estimation showed variations in its effect across regions on different types of conflict risk. Such results are following previous studies showing that the effect of COVID-19 on conflict risk had heterogeneity at the country or city level [7,8]. However, we found consistency in the one-month lagged impact of COVID-19 on conflict risk across regions, featuring a negative impact on non-state and violent conflict risk while a positive effect on the incidence of demonstrations. The first feature coincided with a report from the ACLED Project, which claimed that COVID-19 contributed to a substantial reduction in both armed conflict and deaths [49]. One explanation for this could be more intense global cooperation or the proclamation of “COVID ceasefires” [50,51]. However, in the light of qualitative evidence [7], it seems far more likely that armed groups were constrained in their ability to exercise violence. Examples include lockdowns and border closures (which make rebel movement difficult), a prioritization of the pandemic response by governments and insurgents, and the disruption of supply changes (e.g., for weapons and food). Regarding the finding that COVID-19 increased the possibility of demonstrations, the explanation may lie in the economic downturn and overstretched security forces caused by this pandemic. Economic depression leading to rising unemployment along with lockdown orders on public gatherings have inflamed negative emotions and thus increased the opportunity for people to launch protests and riots [4,52,53]. For instance, in Bangladesh, the recession triggered by the COVID-19 pandemic has reduced the payment of workers, leading to protests from vast numbers of garment workers [54]. There was also news reporting that large numbers of angry protesters have taken up the streets to express their frustration with the lockdown restrictions, especially in the second half of 2021, anti-vaccine protests also gained traction [55,56].

The regional differences detected in our analysis are logical in the context of broader political trends and the results of other studies. In North America, for instance, where a populist movement spearheaded by Donald Trump resulted in strong political polarization of the pandemic management, the impact of the one-month-lagged COVID-19 measure has spurred the incidence of demonstrations (V = 0.01). In contrast, in South America, large social movements occurred in 2019 in countries like Chile, Ecuador, and Venezuela, but COVID-19 restrictions have been forbidding people to protest, leading to a decline in the incidence of demonstrations (V=−0.02) [8]. Likewise, the lagged negative effect of COVID-19 on non-state conflict and violent conflict is rather strong in South Asia (V=−0.04/-0.05) and the Middle East (V=−0.04/-0.03). These are exactly the regions where we know that the Islamic State in Iraq suffered from mobility constraints and economic recession. For example, the Taliban canceled their spring offensive to engage in the pandemic response, and Maoist rebels in India suffered from COVID-19-related food insecurity [57,58].

In addition, we confirmed the impact of COVID-19 on the simulation of conflict risk by including COVID-19 in the conflict risk simulation model. We found that COVID-19 could significantly improve the predictive accuracy of conflict simulations, indicating that the pandemic of COVID-19 has influenced the pattern of conflict to some extent. Thus, it is imperative to include the COVID-19 pandemic in post-2019 conflict simulations to increase precision in forecasting worldwide conflict [38,59].

## 6 Conclusions

We built a large database and ran both SEM and BRT models to completely assess the influence of COVID-19 on conflict risk under climate change in order to determine if and how it affects various forms of conflict risk globally. The present findings confirmed that COVID-19 has a significant impact on conflict risk but with variation across regions and types of conflict risk. However, data indicated that when considering a one-month lag, COVID-19 consistently decreases the possibility of non-state and violent conflict risk while increasing the incidence of demonstrations across regions.

Collectively, this study is one of the first to analyze the impact of COVID-19 on conflict risk at a detailed level by effectively combining a global dataset that covers multiple types of conflict risk and multiple intelligent cognition methods. The following are some advantages that this study has over earlier research. The impact of COVID-19 on conflict risk is first examined in this study on a grid scale, which has a finer data scale and a wider data coverage than earlier studies at regional or national dimensions. More importantly, this study explores the impact of COVID-19 on conflict risk from multiple perspectives by using a variety of intelligent cognitive methods, thus the results obtained from this study are more reliable than those obtained by simple statistical analysis of COVID-19 and conflict risk data.

### 7 Societal implications

Based on a theoretical understanding of how COVID-19 influences the incidence of conflict, our study provided compelling evidence that COVID-19 has a complicated and important impact on several forms of conflict risk both worldwide and regionally. On the one hand, it provides new insight into the field of conflict studies. For example, we should consider the driving effect of COVID-19 on conflict when studying conflict risk, to better analyze the driving mechanism of conflict risk. On the other hand, it provides some inspiration for the implementation of corresponding prevention policies. For instance, a reduction in hostilities one month after a large number of COVID-19 fatalities can open a window for ceasefires or peace talks. Authorities must simultaneously get ready to handle the dual problems of high COVID rates and rising societal unrest and dissent.

## 8 Limitations

It is necessary to point out that there are several possible limitations of our analysis. First, regarding the dataset, this study used a relatively small sample size, and the COVID-19 data mostly relied on self-reported data, which may limit the generalizability and accuracy of the study results. Future research should include more comprehensive datasets with large samples to assess the impact of COVID-19 on conflict risk more accurately. Second, in terms of method, this study used convenience sampling methods and a cross-sectional design, which may introduce bias in the sample and limit the establishment of variable causality. Future studies could fruitfully explore this issue further by conducting random sampling of data to reduce random errors and incorporating more reasonable research methods to explore causal links between variables. Third, this study mainly discussed the conflict risk of COVID-19 in a specific cultural context with a specific theme, without investigating other potential factors that may influence the results, and the results may not be generalized to other cultures or contexts. Future research should consider the comparative analysis on different topics when exploring the impact of COVID-19 on conflict risk.

## Funding

This research is supported and funded by the 10.13039/501100001809National Natural Science Foundation of China (42001238 and 42201497), the Strategic Priority Research Program of the 10.13039/501100002367Chinese Academy of Sciences (XDA19040305), 10.13039/501100012492Youth Innovation Promotion Association (2023000117), and the 10.13039/100010269Wellcome Trust (220211). This study contributes to the CLICCS Cluster of Excellence funded by the German Research Foundation (DFG). For the purpose of open access, the author has applied a CC BY public copyright licence to any Author Accepted Manuscript version arising from this submission.

## Author Contributions

Xiaolan Xie, Mengmeng Hao, Fangyu Ding, Quansheng Ge, Dong Jiang: Conceived and designed the experiments; Performed the experiments; Analyzed and interpreted the data; Wrote the paper. Tobias Ide, David Helman, Jürgen Scheffran: Analyzed and interpreted the data; Wrote the paper. Qian Wang, Yushu Qian, Shuai Chen, Jiajie Wu, Tian Ma: Contributed analysis tools or data; Wrote the paper.

## Data and code availability

The code and data used in the present study are freely available online at https://github.com/Tina-Xi/Code-and-data.

## Declaration of competing interest

The authors declare that they have no known competing financial interests or personal relationships that could have appeared to influence the work reported in this paper
