# Supplementary material for: Exploring the worldwide impact of COVID-19 on conflict risk under climate change
Source: Heliyon. 2023 Jun 10;9(6):e17182. doi: 10.1016/j.heliyon.2023.e17182 (PMC10256592; doi:10.1016/j.heliyon.2023.e17182)
Supplement: Multimedia component 1 [file mmc1.docx]

**Exploring the worldwide impact of COVID-19 on conflict risk under climate change**

Xiaolan Xie ^1,2†^, Mengmeng Hao ^1,2†^, Fangyu Ding ^1,2*^, Tobias Ide^3^, David Helman^4,5^, Jürgen Scheffran^6^, Qian Wang^7^, Yushu Qian ^1,2^, Shuai Chen ^1,2^, Jiajie Wu ^1,2^, Tian Ma ^1,2^, Quansheng Ge ^1*^, Dong Jiang ^1,2*^

^1^ Institute of Geographic Sciences and Natural Resources Research, Chinese Academy of Sciences, Beijing 100101, China.

^2^ College of Resources and Environment, University of Chinese Academy of Sciences, Beijing 100049, China.

^3^ Murdoch University, Murdoch 6150, Perth, Australia.

^4^ Institute of Environmental Sciences, Department of Soil and Water Sciences, The Robert H. Smith Faculty of Agriculture, Food & Environment, The Hebrew University of Jerusalem, Rehovot 7610001, Israel.

^5^ Advanced School for Environmental Studies, The Hebrew University of Jerusalem, Jerusalem, Israel.

^6^ Institute of Geography, Center for Earth System Research and Sustainability, University of Hamburg, Hamburg 20144, Germany.

^7^ Centre for Tropical Medicine, Nuffield Department of Clinical Medicine, University of Oxford, United Kingdom.

† X.L.X. and M.M.H. contributed equally to this work.

* To whom correspondence may be addressed.

Corresponding author: Fangyu Ding (dingfy@igsnrr.ac.cn), Quansheng Ge (geqs@igsnrr.ac.cn) or Dong Jiang (jiangd@igsnrr.ac.cn).

Figures and tables


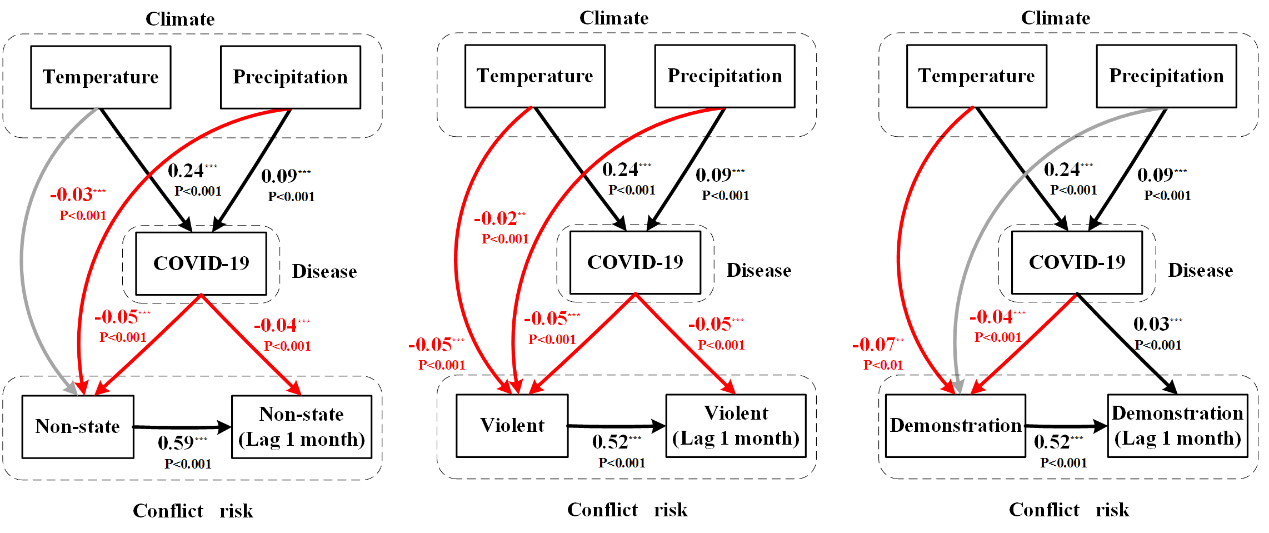


Figure S1. Structural Equation Model in South Asia.


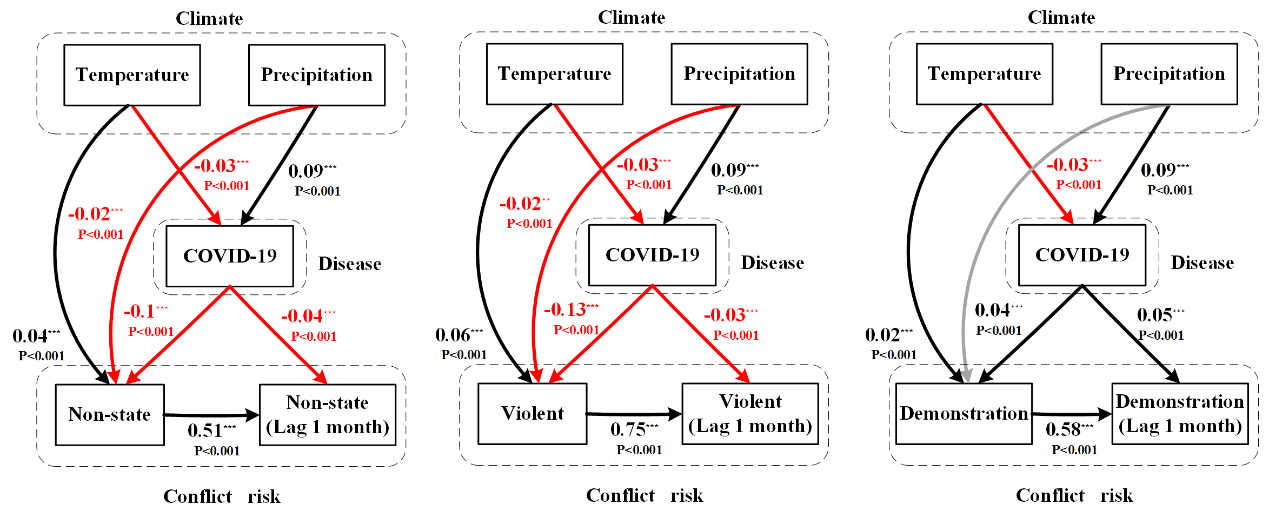


Figure S2. Structural Equation Model in Middle East.


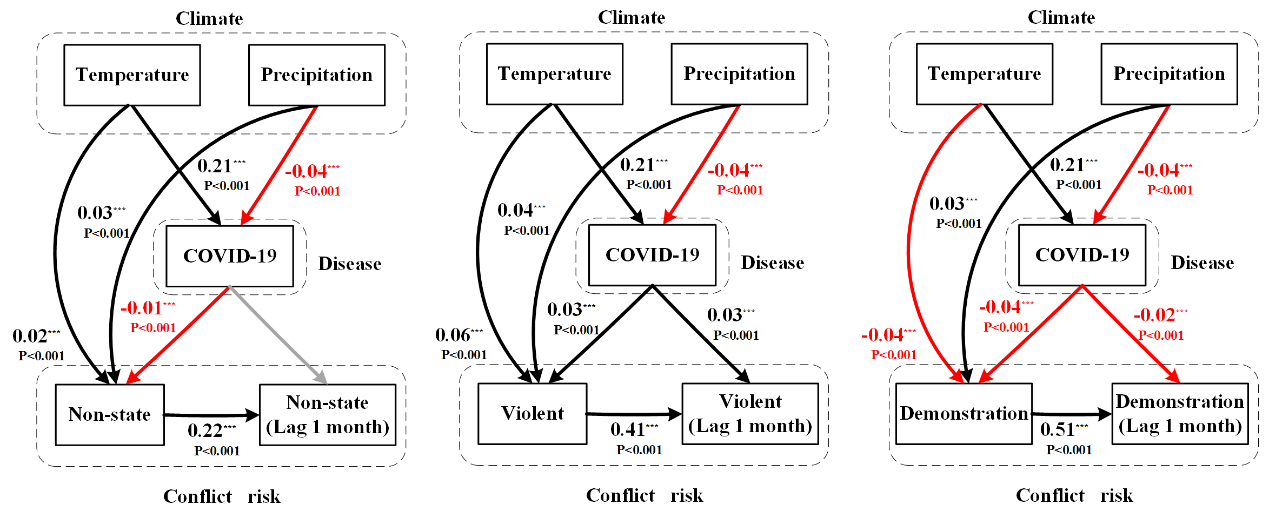


Figure S3. Structural Equation Model in South America.


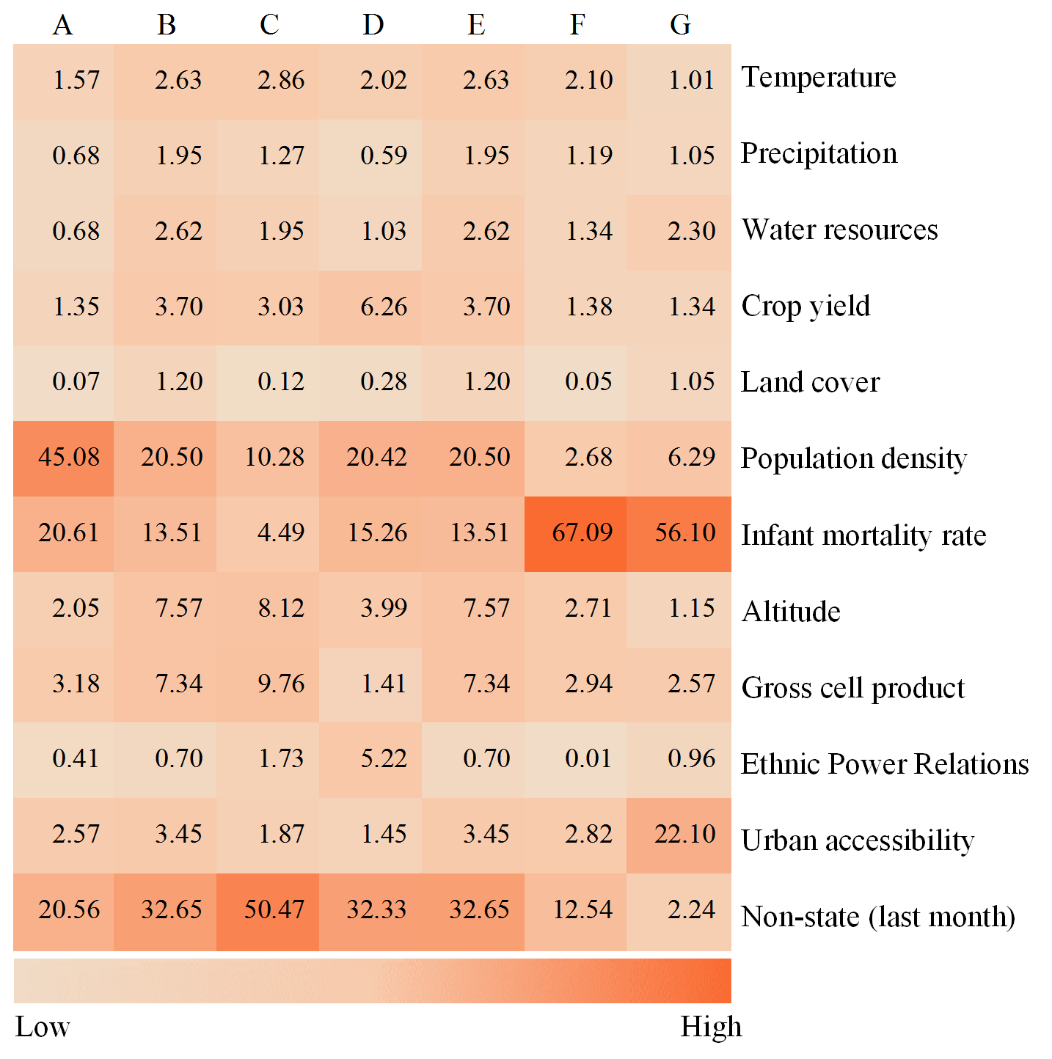


Figure S4. The plot shows the importance of each variable (label in black) on Non-state conflict globally (A), Sub-saharan Africa (B), South Asia (C), Middle East (D), South America (E), North America (F) and Europe (G).


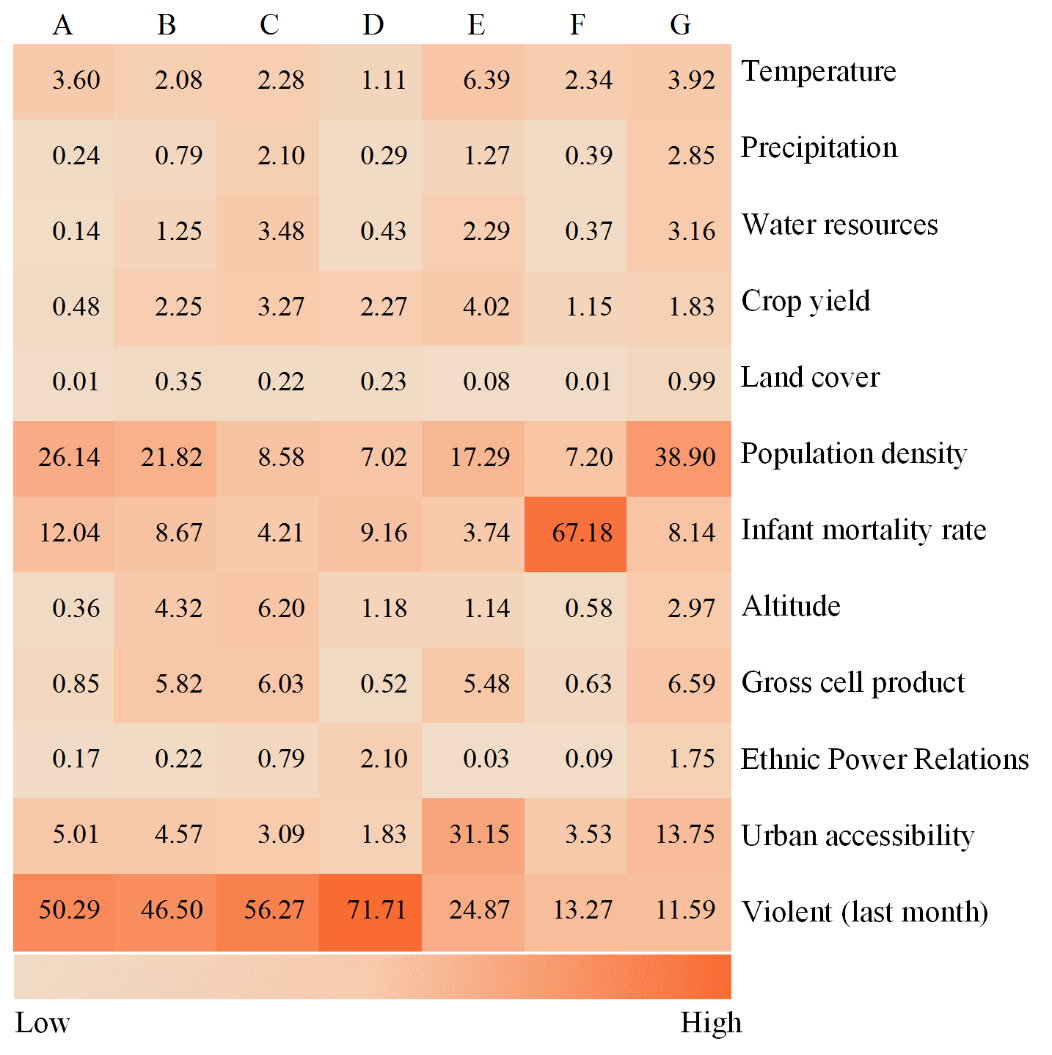


Figure S5. The plot shows the importance of each variable (label in black) on violent conflict globally (A), Sub-saharan Africa (B), South Asia (C), Middle East (D), South America (E), North America (F) and Europe (G).


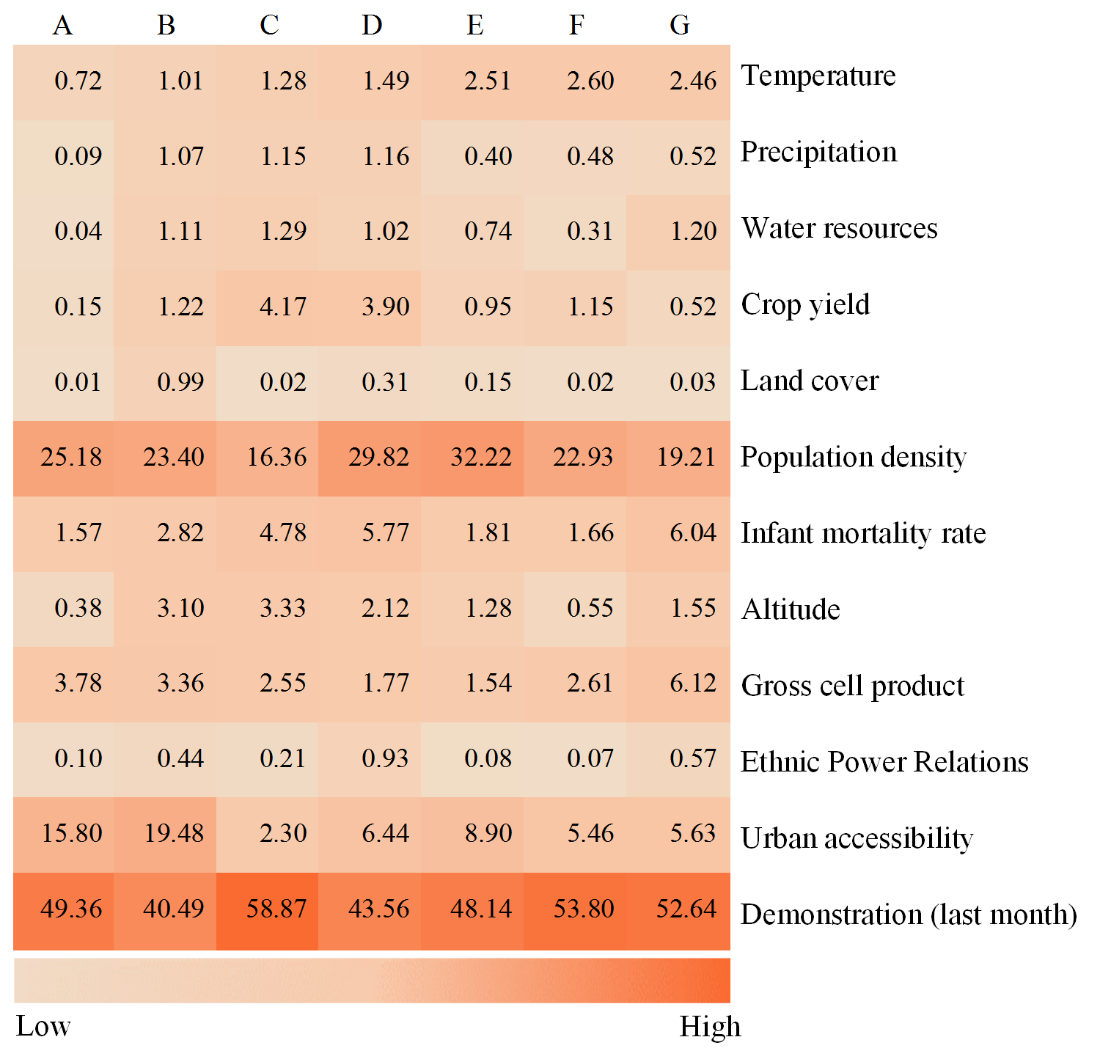


Figure S6. The plot shows the importance of each variable (label in black) on demonstration globally (A), Sub-saharan Africa (B), South Asia (C), Middle East (D), South America (E), North America (F) and Europe (G).

Table S1. List of datasets.

| Indicators | Dataset | Variable | Temporal resolution | Spatial resolution |
| --- | --- | --- | --- | --- |
| Conflict | UCDP GED.v20.1 | Non-state | Month | Single villages |
|  | ACLED | Violent | Month | Single villages |
|  |  | Demonstration | Month | Single villages |
| COVID-19 | Johns Hopkins University | Death toll | Day | Country |
| Climate | MERRA-2 | Temperature | Month | Cell |
|  | ERA5-Land | Precipitation | Month | Cell |
| Environmental background | GES DISC | Soil moisture | Month | Cell |
|  | MODIS | NDVI | Month | Cell |
|  | GPW v4 | Population density | Year | Cell |
|  | SEDAC | Infant mortality rate | Year | Cell |
|  | G-Econ | Gross cell product | Year | Cell |
|  | GlobCover | Land cover | Year | Cell |
|  | SRTM | Altitude | Year | Cell |
|  | GeoEPR | Ethnic Power Relations | Year | Cell |
|  | - | Urban accessibility | Year | Cell |

Table S2. Collinearity statistics in the data. VIF<10 and tolerance >0.1, which indicates no collinearity in the data used in this model.

| Variable | VIF | Tolerance |
| --- | --- | --- |
| COVID-19 | 0.93 | 1.075 |
| Temperature | 0.478 | 2.091 |
| Precipitation | 0.622 | 1.607 |
| Water resources | 0.547 | 1.829 |
| Crop yield | 0.514 | 1.947 |
| Land cover | 0.906 | 1.104 |
| Population density | 0.975 | 1.025 |
| Infant mortality rate | 0.8 | 1.249 |
| Altitude | 0.865 | 1.156 |
| Gross cell product | 0.983 | 1.017 |
| Ethnic Power Relations | 0.854 | 1.171 |
| Urban accessibility | 0.75 | 1.333 |

Table S3. Fit indexes (CFI, NFI, GFI, RMSEA, and SRMR) of the SEM globally.

| Index | Conflict | | | Threshold |
| --- | --- | --- | --- | --- |
|  | Non-state | Violent | Demonstrations |  |
| CFI | 0.969 | 0.967 | 0.964 | ≥0.9 |
| NFI | 0.969 | 0.967 | 0.964 | ≥0.9 |
| GFI | 0.991 | 0.99 | 0.989 | ≥0.9 |
| RMSEA | 0.058 | 0.061 | 0.064 | < 0.1 |
| SRMR | 0.0263 | 0.0277 | 0.0293 | <0.1 |

Table S4. Fit indexes (CFI, NFI, GFI, RMSEA, and SRMR) of the SEM in Sub-saharan Africa.

| Index | Conflict | | | Threshold |
| --- | --- | --- | --- | --- |
|  | Non-state | Violent | Demonstrations |  |
| CFI | 0.98 | 0.978 | 0.973 | ≥0.9 |
| NFI | 0.98 | 0.978 | 0.973 | ≥0.9 |
| GFI | 0.991 | 0.99 | 0.988 | ≥0.9 |
| RMSEA | 0.056 | 0.06 | 0.066 | < 0.1 |
| SRMR | 0.0225 | 0.0246 | 0.0278 | <0.1 |

Table S5. Fit indexes (CFI, NFI, GFI, RMSEA, and SRMR) of the SEM in South Asia.

| Index | Conflict | | | Threshold |
| --- | --- | --- | --- | --- |
|  | Non-state | Violent | Demonstrations |  |
| CFI | 0.979 | 0.979 | 0.976 | ≥0.9 |
| NFI | 0.979 | 0.979 | 0.976 | ≥0.9 |
| GFI | 0.988 | 0.988 | 0.986 | ≥0.9 |
| RMSEA | 0.068 | 0.067 | 0.072 | < 0.1 |
| SRMR | 0.0287 | 0.0282 | 0.0338 | <0.1 |

Table S6. Fit indexes (CFI, NFI, GFI, RMSEA, and SRMR) of the SEM in Middle East.

| Index | Conflict | | | Threshold |
| --- | --- | --- | --- | --- |
|  | Non-state | Violent | Demonstrations |  |
| CFI | 0.908 | 0.924 | 0.912 | ≥0.9 |
| NFI | 0.908 | 0.923 | 0.912 | ≥0.9 |
| GFI | 0.966 | 0.965 | 0.966 | ≥0.9 |
| RMSEA | 0.116 | 0.118 | 0.116 | < 0.1 |
| SRMR | 0.0516 | 0.0503 | 0.053 | <0.1 |

Table S7. Fit indexes (CFI, NFI, GFI, RMSEA, and SRMR) of the SEM in South America.

| Index | Conflict | | | Threshold |
| --- | --- | --- | --- | --- |
|  | Non-state | Violent | Demonstrations |  |
| CFI | 0.984 | 0.98 | 0.982 | ≥0.9 |
| NFI | 0.984 | 0.98 | 0.981 | ≥0.9 |
| GFI | 0.993 | 0.991 | 0.992 | ≥0.9 |
| RMSEA | 0.049 | 0.056 | 0.055 | < 0.1 |
| SRMR | 0.0179 | 0.0212 | 0.0211 | <0.1 |

Table S8. Fit indexes (CFI, NFI, GFI, RMSEA, and SRMR) of the SEM in North America.

| Index | Conflict | | | Threshold |
| --- | --- | --- | --- | --- |
|  | Non-state | Violent | Demonstrations |  |
| CFI | 0.968 | 0.965 | 0.959 | ≥0.9 |
| NFI | 0.968 | 0.965 | 0.959 | ≥0.9 |
| GFI | 0.988 | 0.986 | 0.984 | ≥0.9 |
| RMSEA | 0.065 | 0.072 | 0.076 | < 0.1 |
| SRMR | 0.027 | 0.03 | 0.0325 | <0.1 |

Table S9. Fit indexes (CFI, NFI, GFI, RMSEA, and SRMR) of the SEM in Europe.

| Index | Conflict | | | Threshold |
| --- | --- | --- | --- | --- |
|  | Non-state | Violent | Demonstrations |  |
| CFI | 0.956 | 0.949 | 0.944 | ≥0.9 |
| NFI | 0.956 | 0.949 | 0.944 | ≥0.9 |
| GFI | 0.984 | 0.983 | 0.978 | ≥0.9 |
| RMSEA | 0.078 | 0.08 | 0.09 | < 0.1 |
| SRMR | 0.0341 | 0.0351 | 0.0401 | <0.1 |

Table S10. Summary effect of climate (temperature and precipitation) on COVID-19 at global and regional scale. **P < 0.01; ***P < 0.001.

| Region | Climate-> COVID-19 | |
| --- | --- | --- |
|  | Temperature | Precipitation |
| Global | -0.1^***^ | 0.08^***^ |
| Sub-saharan Africa | -0.3^***^ | -0.07^***^ |
| South Asia | 0.24^***^ | 0.09^***^ |
| Middle East | -0.03^***^ | 0.09^***^ |
| South America | 0.21^***^ | -0.04^***^ |
| North America | -0.32^***^ | -0.01^**^ |
| Europe | -0.14^***^ | -0.09^***^ |

Table S11. Effect of variables on three types of conflict at global scale. NS: not significant, P>0.05; *P < 0.05; **P < 0.01; ***P< 0.001.

| Variable | Non-state | Violent | Demonstrations | |
| --- | --- | --- | --- | --- |
| Temperature | 0.019^***^ | 0.049^***^ | | 0.036^***^ |
| Precipitation | NS | 0.013^***^ | | NS |
| Water resources | 0.018^***^ | 0.03^***^ | | 0.028^***^ |
| Crop yield | -0.035^***^ | -0.029^***^ | | 0.033^***^ |
| Land cover | 0.024^***^ | 0.049^***^ | | 0.106^***^ |
| Population density | 0.022^***^ | 0.063^***^ | | 0.154^***^ |
| Infant mortality rate | 0.115^***^ | 0.147^***^ | | -0.008^***^ |
| Altitude | 0.028^***^ | 0.016^***^ | | 0.008^***^ |
| Gross cell product | 0.002^*^ | NS | | 0.09^***^ |
| Ethnic Power Relations | 0.02^***^ | 0.023^***^ | | 0.007^***^ |
| Urban accessibility | -0.033^***^ | -0.049^***^ | | -0.08^***^ |

Table S12. Effect of variables on three types of conflict in Sub-saharan Africa. NS: not significant, P>0.05; *P < 0.05; **P < 0.01; ***P< 0.001.

| Variable | Non-state | Violent | Demonstrations |
| --- | --- | --- | --- |
| Temperature | 0.034^***^ | 0.047^***^ | NS |
| Precipitation | 0.053^***^ | 0.039^***^ | 0.019^***^ |
| Water resources | -0.024^***^ | 0.009^*^ | 0.012^*^ |
| Crop yield | -0.033^***^ | -0.055^***^ | NS |
| Land cover | 0.06^***^ | 0.082^***^ | 0.062^***^ |
| Population density | 0.069^***^ | 0.118^***^ | 0.207^***^ |
| Infant mortality rate | 0.073^***^ | 0.093^***^ | -0.021^***^ |
| Altitude | 0.029^***^ | NS | -0.02 |
| Gross cell product | -0.007^**^ | 0.007^**^ | 0.066^***^ |
| Ethnic Power Relations | -0.019^***^ | -0.015^***^ | 0.023^*^ |
| Urban accessibility | -0.051^***^ | -0.092^***^ | -0.065^***^ |

Table S13. Effect of variables on three types of conflict in South Asia. NS: not significant, P>0.05; *P < 0.05; **P < 0.01; ***P< 0.001.

| Variable | Non-state | Violent | Demonstrations |
| --- | --- | --- | --- |
| Temperature | NS | -0.045^***^ | -0.073^***^ |
| Precipitation | -0.027^***^ | -0.019^**^ | NS |
| Water resources | 0.068^***^ | 0.062^***^ | 0.026^***^ |
| Crop yield | -0.14^***^ | -0.094^***^ | 0.146^***^ |
| Land cover | NS | NS | 0.04^***^ |
| Population density | -0.012^*^ | 0.053^***^ | 0.129^***^ |
| Infant mortality rate | 0.03^***^ | 0.055^***^ | -0.039^***^ |
| Altitude | 0.199^***^ | 0.125^***^ | 0.105^***^ |
| Gross cell product | -0.061^***^ | -0.048^***^ | 0.045^***^ |
| Ethnic Power Relations | 0.151^***^ | 0.131^***^ | -0.011^*^ |
| Urban accessibility | -0.165^***^ | -0.155^***^ | -0.04^***^ |

Table S14. Effect of variables on three types of conflict in the Middle East. NS: not significant, P>0.05; *P < 0.05; **P < 0.01; ***P< 0.001.

| Variable | Non-state | Violent | Demonstrations |
| --- | --- | --- | --- |
| Temperature | 0.038^***^ | 0.055^***^ | 0.02^***^ |
| Precipitation | -0.02^***^ | -0.015^**^ | NS |
| Water resources | 0.042^***^ | 0.058^***^ | 0.053^***^ |
| Crop yield | 0.076^***^ | 0.116^***^ | 0.105^***^ |
| Land cover | 0.097^***^ | 0.09^***^ | 0.129^***^ |
| Population density | 0.048^***^ | 0.067^***^ | 0.124^***^ |
| Infant mortality rate | 0.171^***^ | 0.295^***^ | 0.063^***^ |
| Altitude | -0.038^***^ | -0.053^***^ | -0.05^***^ |
| Gross cell product | -0.03^***^ | -0.037^***^ | NS |
| Ethnic Power Relations | 0.162^***^ | 0.225^***^ | 0.129^***^ |
| Urban accessibility | NS | -0.02^***^ | -0.05^***^ |

Table S15. Effect of variables on three types of conflict in South America. NS: not significant, P>0.05; *P < 0.05; **P < 0.01; ***P< 0.001.

| Variable | Non-state | Violent | Demonstrations |
| --- | --- | --- | --- |
| Temperature | 0.024^***^ | 0.059^***^ | -0.037^***^ |
| Precipitation | 0.032^***^ | 0.042^***^ | 0.029^***^ |
| Water resources | NS | 0.021^***^ | -0.011^*^ |
| Crop yield | 0.023^***^ | 0.027^***^ | 0.025^***^ |
| Land cover | 0.012^***^ | 0.05^***^ | 0.049^***^ |
| Population density | 0.016^***^ | 0.073^***^ | 0.113^***^ |
| Infant mortality rate | -0.017^***^ | -0.022^***^ | 0.012^***^ |
| Altitude | 0.031^***^ | 0.033^***^ | 0.024^***^ |
| Gross cell product | 0.008^**^ | 0.098^***^ | 0.066^***^ |
| Ethnic Power Relations | 0.014^***^ | 0.014^***^ | 0.02^***^ |
| Urban accessibility | -0.031^***^ | -0.115^***^ | -0.112^***^ |

Table S16. Effect of variables on three types of conflict in North America. NS: not significant, P>0.05; *P < 0.05; **P < 0.01; ***P< 0.001.

| Variable | Non-state | Violent | Demonstrations |
| --- | --- | --- | --- |
| Temperature | 0.027^***^ | 0.039^***^ | 0.045^***^ |
| Precipitation | -0.021^***^ | NS | NS |
| Water resources | NS | 0.02^***^ | 0.064^***^ |
| Crop yield | NS | 0.035^***^ | 0.078^***^ |
| Land cover | NS | 0.023^***^ | NS |
| Population density | 0.074^***^ | 0.137^***^ | 0.182^***^ |
| Infant mortality rate | 0.132^***^ | 0.243^***^ | -0.022^***^ |
| Altitude | 0.096^***^ | 0.082^***^ | -0.038^***^ |
| Gross cell product | 0.012^***^ | -0.011^***^ | 0.118^***^ |
| Ethnic Power Relations | 0.033^***^ | 0.009^**^ | -0.02^***^ |
| Urban accessibility | -0.011^*^ | -0.03^***^ | -0.134^***^ |

Table S17. Effect of variables on three types of conflict in Europe. NS: not significant, P>0.05; *P < 0.05; **P < 0.01; ***P< 0.001.

| Variable | Non-state | Violent | Demonstrations |
| --- | --- | --- | --- |
| Temperature | 0.01^*^ | 0.028^***^ | -0.016^***^ |
| Precipitation | -0.007^*^ | NS | 0.039^***^ |
| Water resources | NS | 0.009^*^ | -0.058^***^ |
| Crop yield | NS^*^ | -0.018^**^ | 0.071^***^ |
| Land cover | 0.03^***^ | 0.041^***^ | 0.079^***^ |
| Population density | NS | 0.076^***^ | 0.168^***^ |
| Infant mortality rate | 0.041^***^ | 0.05^***^ | -0.126^***^ |
| Altitude | NS | NS | 0.03^***^ |
| Gross cell product | NS | NS | 0.122^***^ |
| Ethnic Power Relations | 0.01^**^ | 0.012^***^ | -0.022^***^ |
| Urban accessibility | -0.011^**^ | -0.019^***^ | -0.11^***^ |
